# Books and Pamphlets Received

**Published:** 1887-07

**Authors:** 


### BOOKS AND PAMPHLETS RECEIVED.

*A Manual of Practical Therapeutics*, considered with reference to articles of the Materia Medica. By Edward John Waring, C. I. E, M. D., Fellow of the Royal College of Physicians, London; Surgeon-Major in Her Majesty's Indian Army. Edited by Dudley W. Buxton, M. D, B. S., London, Member of the Royal College of Physicians; Assistant to the Professor of Medicine at University College, London, and Administrator of Anæsthetics at University College Hospital and the Dental Hospital of London. Fourth edition. Philadelphia: P. Blakiston, Son & Co. 1886.

The above work contains 657 pages of condensed useful practical matter, in which the therapeutics of all important drugs is given, including new remedies and recent advances in this line, down to the latest moment. It is an exceedingly valuable book.

*The Century Magazine*.—The ability and excellence of this great magazine is well mantained in the last or June number.

The Lincoln History makes marked progress toward the presidential contest of 1860 by the consideration of events which form a background to a proper presentation of Lincoln's personality, events also which are necessary to an understanding of his personal career. The special topics in the present part are the attack on Charles Sumner by Preston S. Brooks, and the Dred Scott decision of March 9, 1857. These events are rapidly and clearly sketched, and Lincoln's views of the decision recorded in his own words. The portraits include Sumner, Brooks, Henry Wilson, Anson Burlingame, Dred Scott and his wife, Chief-Justice Taney, and Associate Justices McLean, Nelson, and Curtis. This record of the circumstances under which the decision was made has the advantage that one of the authors, Mr. Nicolay, has been for years the Marshal of the Supreme Court of the United States, and thus has had special facilities for studying the subject.

A thoughtful and suggestive paper by the Rev. T. T. Munger, considering the true aim and the best methods of education, bears the title, "Educational and Social Progress." It is in the nature of a protest against the tendency to specialization and false utilitarianism in contemporary college instruction.

The battle series makes a comprehensive advance with a paper by General E. M. Law on the Virginia campaign of 1864, entitled "From the Wilderness to Cold Harbor," those two engagements and the intermediate ones of Spotsylvania Court House and the North Anna being included in a popular record richly supplemented with illustrations from sketches made at the time, and with official maps. A short article is devoted to the "Hand-to-Hand Fighting at Spotsylvania," perhaps the most stubborn contest of the war. The writer is Mr. G. Norton Galloway, historian of the Sixth Corps. *The Century* also makes public for the first time a complete resume of the aggregate forces and losses of the Union army in the Wilderness campaign. Short supplementary "Memoranda" are contributed by General Thomas Jordan on "A Missing Cipher Dispatch," and by General R. E. Colston on "Union Sentiment among Confederate Veterans." There is also an editorial article on "Lord Wolseley's Estimate of General Lee," taking note of the numerous and fundamental historical inaccuracies of General Wolsey's recent essay.

Mr. Stockton's serial, "The Hundredth Man," is continued; and Miss Elizabeth Stuart Phelps contributes a tragic and heartrending story, entitled "Jack," which is illustrated by Irving R. Wiles and Mrs. Mary Hallock Foote. "Jack" is a fisherman, who inherits the tendency to drink; he is a typical case, whose study is recommended to all interested in the temperance cause.

The poetry includes a collection of five "Songs of the Sea," by William Sterne ("Solitude"—"Silence"), Charles Edwin Markham ("After Reading Shakspere"), and James Whitcom Riley ("When She Comes Home"); and a lyric by George Parsons Lathrop, entitled "The Name of Washington."

A second editorial article is entitled "Landscape Gardeners Needed for America." The "Open Letters" are on "Church Union." "From an Unitarian Point of View," by the Revs. Edward Everett Hale and Andrew P. Peabody; "Applause as a Spur to Pegasus, by Rev. William C. Wilkinson, and other communications on "John Tyler," "The Cosmic Day," etc." In "Bric-a-Brac" are poems by Marian Douglas, Ernest Whitney, Margaret Deland, Margaret Vandegrift, and Frances Hodgson Burnett ("Point d'Alencon").

